# Poorly controlled diabetes during pregnancy and lactation activates the Foxo1 pathway and causes glucose intolerance in adult offspring

**DOI:** 10.1038/s41598-019-46638-2

**Published:** 2019-07-15

**Authors:** Yukihiro Inoguchi, Kenji Ichiyanagi, Hiroaki Ohishi, Yasutaka Maeda, Noriyuki Sonoda, Yoshihiro Ogawa, Toyoshi Inoguchi, Hiroyuki Sasaki

**Affiliations:** 1 Division of Epigenomics and Development, Department of Molecular and Structural Biology Medical Institute of Bioregulation, Kyushu University, Fukuoka, Japan; 2 Department of Medicine and Bioregulatory Science, Graduate School of Medical Sciences, Kyushu University, Fukuoka, Japan; 3 Laboratory of Genome and Epigenome Dynamics, Department of Animal Sciences, Graduate School of Bioagricultural Sciences, Nagoya University, Nagoya, Japan; 4 Clinical Research Center for Diabetes, Clinic Masae Minami, Fukuoka, Japan; 5 Department of Molecular and Cellular Metabolism, Graduate School of Medical and Dental Sciences, Tokyo Medical and Dental University, Tokyo, Japan; 6 AMED-CREST, Japan Agency for Medical Research and Development, Tokyo, Japan; 7 Fukuoka Health Promotion Support Center, Fukuoka, Japan

**Keywords:** Diabetes, Diabetes

## Abstract

Exposure to maternal diabetes during pregnancy results in diabetes in offspring, but its underlying mechanisms are unclear. Here, we investigated the phenotype and molecular defects of the offspring of poorly controlled diabetic female mice generated by streptozotocin (STZ) administration. Offspring was exposed to maternal diabetes during pregnancy and lactation. The body weight of STZ offspring was lower than that of control offspring at birth and in adulthood, and glucose tolerance was impaired in adult STZ offspring. Interestingly, the phenotype was more pronounced in male offspring. We next investigated the morphology of islets and expression of β cell-related genes, but no significant changes were observed. However, transcriptome analysis of the liver revealed activation of the fork head box protein O1 (Foxo1) pathway in STZ male offspring. Notably, two key gluconeogenesis enzyme genes, glucose 6 phosphatase catalytic subunit (*G6pc*) and phosphoenolpyruvate carboxykinase 1 (*Pck1*), were upregulated. Consistent with this finding, phosphorylation of Foxo1 was decreased in the liver of STZ male offspring. These changes were not obvious in female offspring. The activation of Foxo1 and gluconeogenesis in the liver may have contributed to the impaired glucose tolerance of STZ male offspring.

## Introduction

Type 2 diabetes has strong familial aggregation, and genetic studies have identified genes responsible for the increased risk of the disease. However, common variants explain only ~10% of the heritability of type 2 diabetes^1^. Although rare variants with larger effects have been identified, their contribution to the missing heritability is limited at the population level. However, early life environment is implicated in disease development during adulthood^2^, and recent studies suggest an important role of intrauterine exposure to diabetes in the pathogenesis of type 2 diabetes^3–8^. For example, epidemiological studies have revealed higher prevalence of diabetes in the offspring of diabetic women and a greater transmission frequency of the disease from mothers than from fathers^5–7^. High prevalence of type 2 diabetes or the prediabetic state has also been reported in the offspring of women with gestational diabetes or type 1 diabetes^4^. Thus, early exposure to diabetes may in part explain the missing heritability of type 2 diabetes.

Animal models have also shown a relationship between maternal diabetes and diabetic phenotypes of offspring. For example, rats or mice injected with streptozotocin (STZ), infused with glucose, or mutated for the leptin receptor show that the diabetic condition during pregnancy leads to impaired glucose tolerance (IGT), impaired insulin secretion, and/or increased insulin resistance in adult offspring^9–14^. Thus, the animal studies support the results of human studies and indicate an effect of the intrauterine diabetic environment on the risk of developing type 2 diabetes. Furthermore, studies have suggested that offspring may have impaired insulin secretion^10,11,13^ reduced glucose uptake in peripheral tissues^12^, and/or increased gluconeogenesis in the liver^12,13^. Understanding the precise mechanisms of these events may be important to identify specific risk groups and develop strategies to prevent type 2 diabetes in future generations.

In the present study, we investigated the possible mechanisms underlying the IGT observed in adult offspring of poorly controlled STZ-induced diabetic mice. We found that the Foxo1 pathway is one signalling pathway affected by maternal diabetes in male offspring. Dysregulation of Foxo1 target genes in the liver may contribute to increased gluconeogenesis in the liver, eventually leading to IGT.

## Results

### Effect of maternal diabetes on litter size, body weight, and organ weight

**Figure 1:**
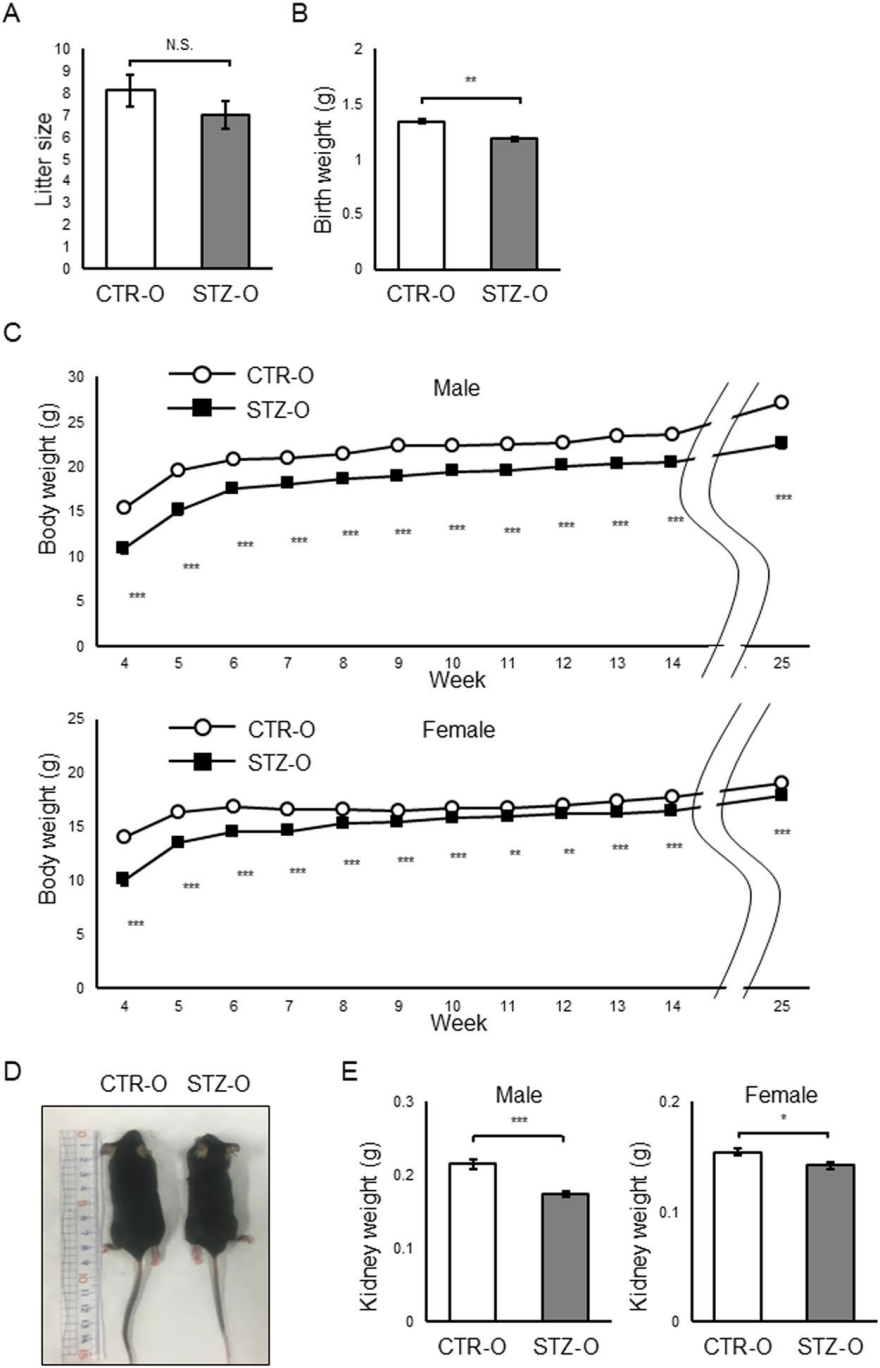
Body and organ weights of STZ offspring. (**A**) Litter size (n = 10 for each CTR and STZ offspring) (**B**) Birth weight (CTR offspring, n = 35; STZ offspring, n = 27) (**C**) Changes in body weight during postnatal development. Upper panel, males (CTR offspring, n = 20; STZ offspring, n = 16); lower panel, females (CTR offspring, n = 27; STZ offspring, n = 23). (**D**) Representative image of CTR and STZ male offspring at 25 weeks. (**E**) Kidney weight at 26–27 weeks. Left, males (CTR offspring, n = 12; STZ offspring, n = 12); right, females (CTR offspring, n = 13; STZ offspring, n = 14). CTR-O, CTR offspring; STZ-O, STZ offspring. Data represent the mean ± SE. *P < 0.05, **P < 0.01, ***P < 0.001, N.S. not significant.

We examined the litter size and body weight of newborn pups from STZ-induced diabetic female mice (STZ offspring). While the litter size was not significantly different from that of the control (CTR) (Fig. 1A), the proportion of STZ offspring that died before weaning (17.1%, 12/70) was significantly higher than that of CTR offspring (3.7%, 3/81) (P < 0.01). Also, the body weight of STZ offspring was clearly lower at birth (Fig. 1B), and it persisted to adulthood in both male and female offspring, although the difference was larger in males (Fig. 1C,D). We also found that the kidney weight was reduced in STZ offspring at 26–27 weeks (Fig. 1E). Again, the difference was more significant in males. These results indicate that exposure to poorly controlled maternal diabetes during pregnancy and lactation has a long-term effect on body and organ weights of the offspring with a sex difference. The reduced body weight has also been reported in previous animal models^10,12^ but this phenotype is not common to all models.

### Effect of maternal diabetes on glucose tolerance

**Figure 2:**
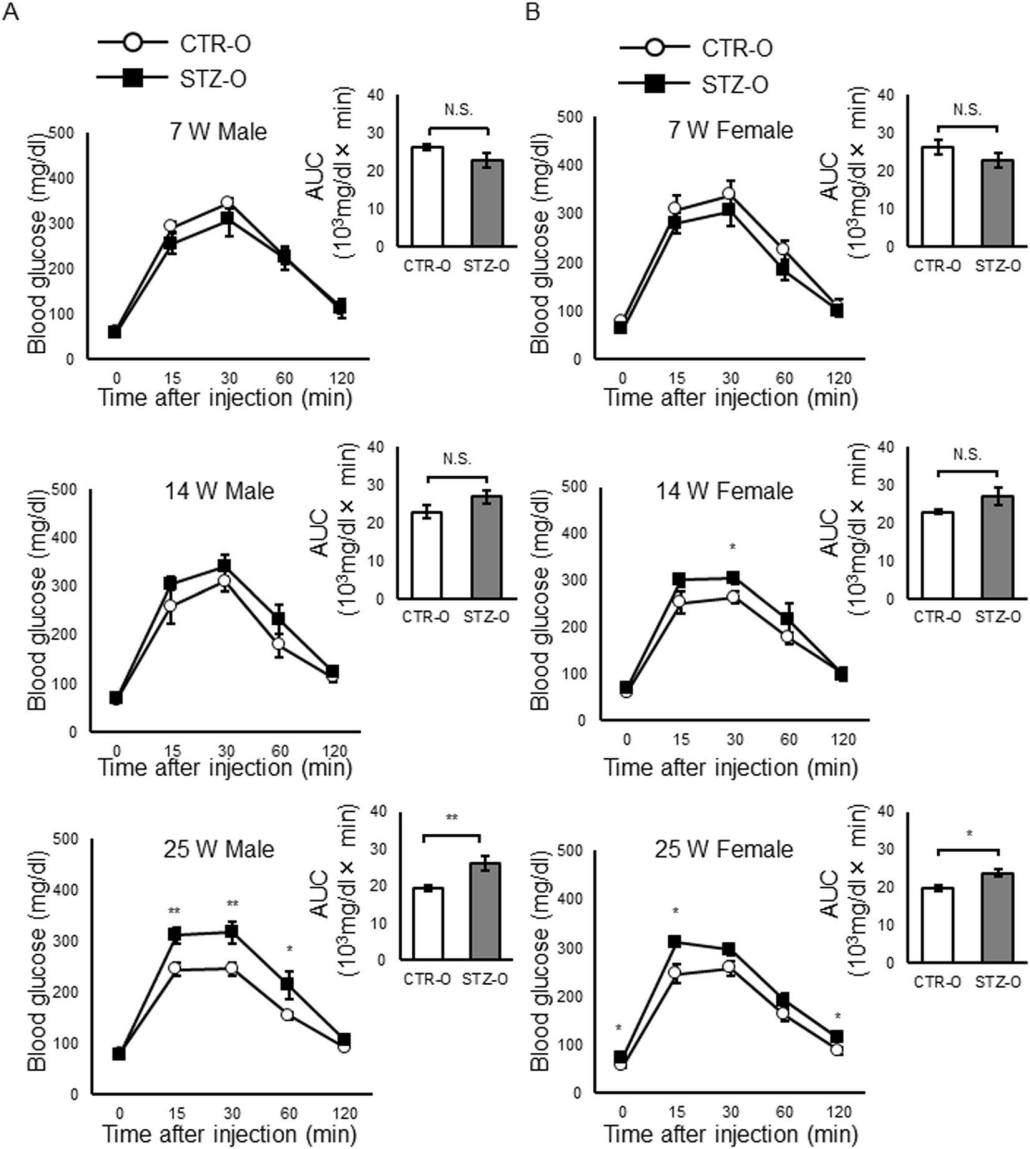
IPGTT. (**A**,**B**) Blood glucose levels during the IPGTT (left) and areas under the curve (AUC) (right) in male (**A**) and female offspring (**B**) at 7, 14, and 25 weeks. Blood glucose levels were measured after intraperitoneal injection of glucose (1 g/kg body weight). The numbers of male animals used in (**A**) are: 7 weeks, CTR offspring, n = 7 and STZ offspring, n = 6; 14 weeks, CTR offspring, n = 7 and STZ offspring, n = 6; 25 weeks, CTR offspring, n = 7 and STZ offspring, n = 6. The numbers of female animals used in (**B**) are: 7weeks, CTR offspring, n = 7 and STZ offspring, n = 6; 14 weeks, CTR offspring, n = 7 and STZ offspring, n = 6; 25 weeks, CTR offspring, n = 6 and STZ offspring, n = 6). Data represent the mean ± SE. *P < 0.05, **P < 0.01, N.S. not significant.

To investigate the glucose metabolism in STZ offspring, we performed an intraperitoneal glucose tolerance test (IPGTT) (Fig. 2A,B). At 7 weeks, there was no significant difference in glucose tolerance between STZ and CTR offspring. However, at 14 weeks, the blood glucose level was slightly higher in STZ offspring, although the difference was significant only at 30 min after glucose injection in females. At 25 weeks, the area under the blood glucose level-time curve (AUC) was significantly larger in STZ offspring regardless of sex. However, the difference was more significant in males at multiple time points. These results suggest that the exposure to maternal diabetes during pregnancy and lactation causes IGT in offspring, as reported previously^9,11,13^.

### Effect of maternal diabetes on islets and insulin secretion

To understand the mechanism underlying the IGT, we first measured the fasting serum insulin level and area dimension of pancreatic islets. We also immunostained pancreatic tissues for insulin and measured the intensity. No significant difference was found in either of the above between STZ and CTR offspring at 25–27 weeks (Supplementary Fig. 1A–C). We also examined the expression of genes involved in islet development and functions by quantitative reverse transcription-polymerase chain reaction (qRT-PCR) in islets isolated from the offspring at 18 weeks. There was no significant difference in the expression of most examined genes, including hepatocyte nuclear factor 1α (*Hnf1α*), *Hnf4α7*, solute carrier family 2 member 2 (*Scl2a2*), insulin 1 (*Ins1*), and *Ins2* (Supplementary Fig. 1D), regardless of sex. Only pancreatic and duodenal homeobox 1 (*Pdx1*), a transcription factor necessary for pancreatic development and β cell maturation, showed higher expression in STZ female offspring (Supplementary Fig. 1D).

### Effect of maternal diabetes on the transcriptome in the liver

**Figure 3:**
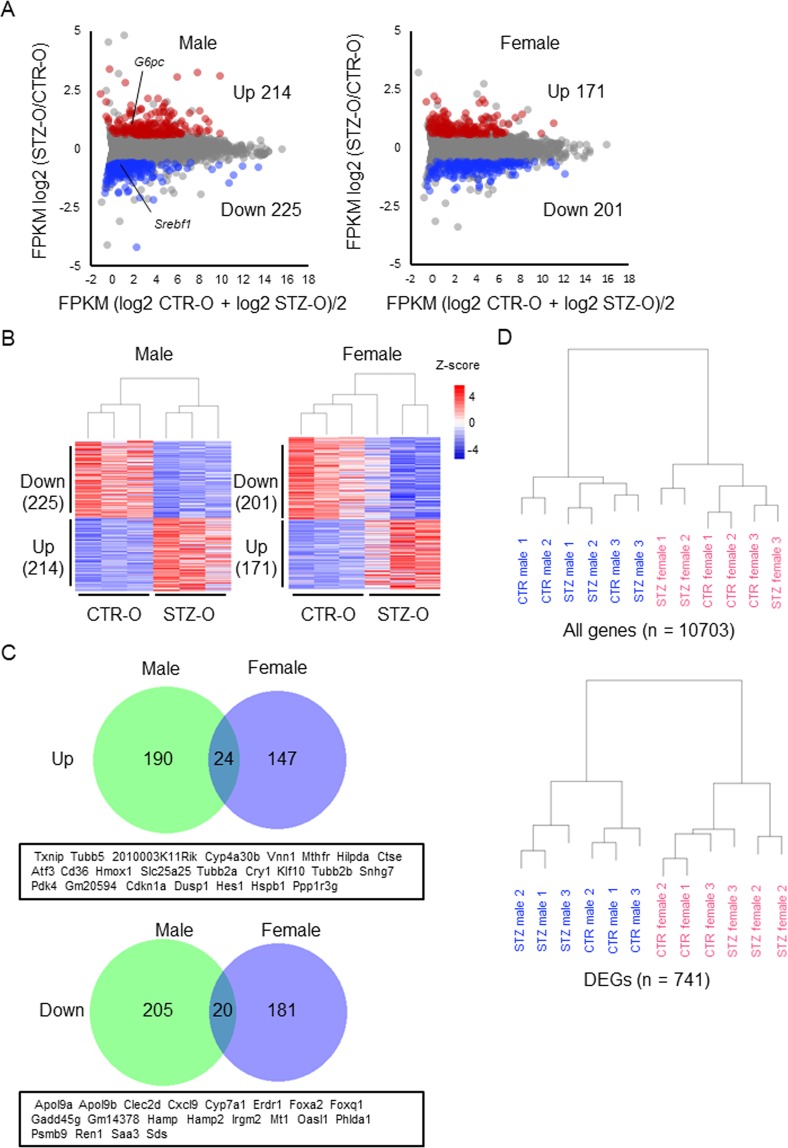
Transcriptome analysis of the liver. (**A**) MA plots of gene expression changes in the liver examined by mRNA-seq. Liver samples were collected from CTR and STZ offspring at 26–27 weeks. Expression levels are indicated in FPKM. Red dots indicate upregulated DEGs and the blue dots indicate downregulated DEGs. (**B**) Z-score-normalised heatmaps of DEGs. Three independent samples were analyzed (n = 3) for the respective sex and category (CTR or STZ offspring). Statistical differences were calculated using the cuffdiff program^46^ (**C**) Venn diagrams showing overlaps between upregulated DEGs of males and females (upper panel) and between downregulated DEGs of males and females (lower panel). Diagrams were drawn using BioVenn^47^ (**D**) Cluster analysis of all genes (n = 10703) (upper panel) and DEGs (n = 741) (lower panel). Clusters were determined by R (https://www.r-project.org/) based on the expression levels [Log_2_(FPKM + 1)].

Several studies have shown that the liver is an organ involved in IGT of animals exposed to a diabetic environment or altered nutrient availability in utero^13,15^. Other models also suggest a role of the liver in insulin resistance^12,16^. To determine the effect of maternal diabetes on the transcriptome of the liver, we performed messenger RNA-sequencing (mRNA-seq) of liver samples from STZ and CTR offspring at 26–27 weeks. We found that 214 and 171 genes were significantly upregulated in STZ male and female offspring, respectively (FDR < 0.05 and STZ/CTR > 1.5) (Fig. 3A,B) (Supplementary Data). In addition, 225 and 201 genes were significantly downregulated in STZ male and female offspring, respectively (FDR < 0.05 and CTR/STZ > 1.5). However, when we compared differentially expressed genes (DEGs) identified in male offspring with those in female offspring, only a small number of DEGs overlapped (Fig. 3C), suggesting a sex-dependent effect of maternal diabetes on the transcriptome of the liver. Therefore, the gene expression profiles of individual samples were subjected to cluster analysis using data from all genes that met our criteria (including non-DEGs, n = 10703) (see Methods). The analysis revealed two major clusters, namely male and female clusters (Fig. 3D), suggesting that a sex difference, rather than maternal diabetes, has a stronger effect on gene expression in the liver. Next, we used the gene expression data of all DEGs (n = 741) for cluster analysis and found that the DEG expression profiles were also divided into male and female clusters (Fig. 3D). These results suggested that the DEGs should be separately characterised in male and female offspring.

**Figure 4:**
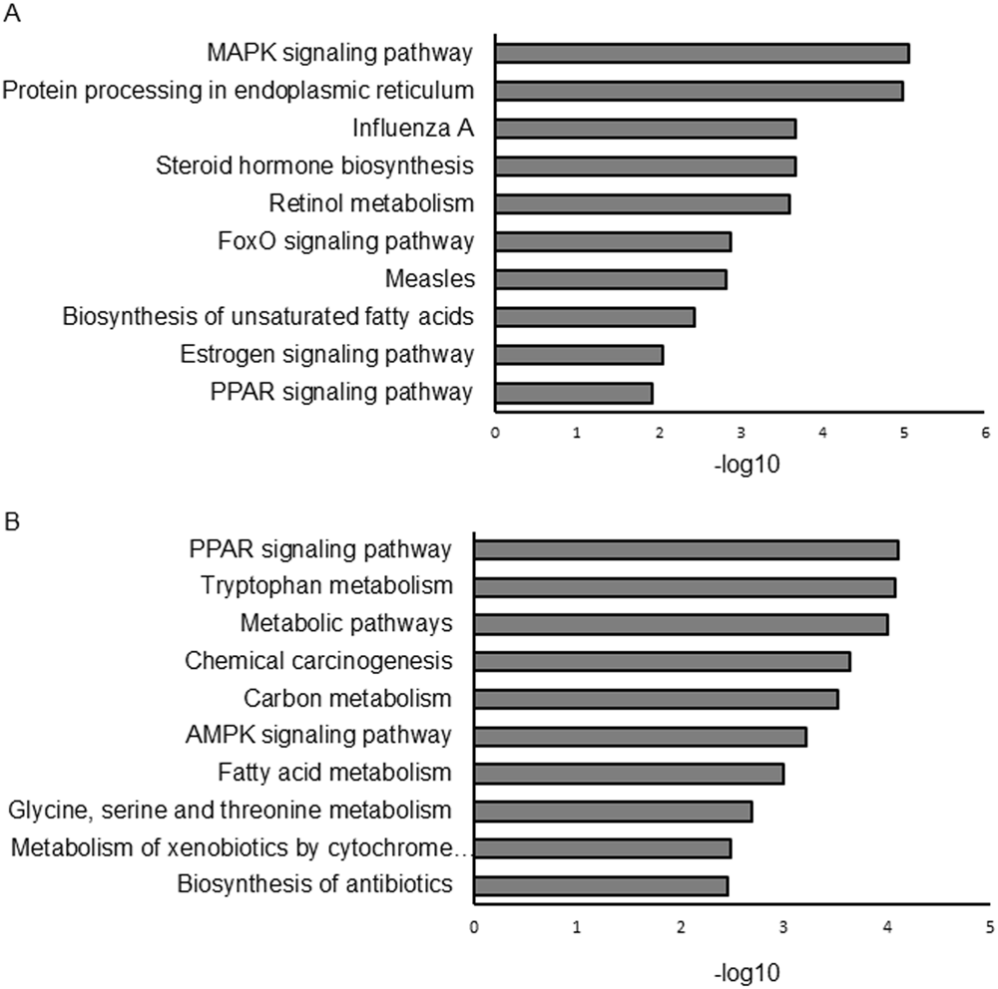
KEGG pathway analysis. Results were shown for DEGs of males (**A**) and females (**B**). DAVID 6.8 (https://david.ncifcrf.gov/) was used for the analysis.

To investigate the biological features of the DEGs, we performed Kyoto Encyclopedia of Genes and Genomes (KEGG) pathway analyses^17^. We found that pathways involved in nutrient metabolism, such as the “forkhead box-containing protein, O sub-family (Foxo) signaling pathway”, and “peroxisome proliferator-activated receptor (PPAR) signaling pathway”, were enriched in the DEGs of male offspring (Fig. 4A), and those such as the “PPAR signaling pathway”, “AMP-activated protein kinase (AMPK) signaling pathway”, and “fatty acid metabolism pathway” were enriched in the DEGs of female offspring (Fig. 4B).

### Expression of the Foxo1 pathway genes

**Figure 5:**
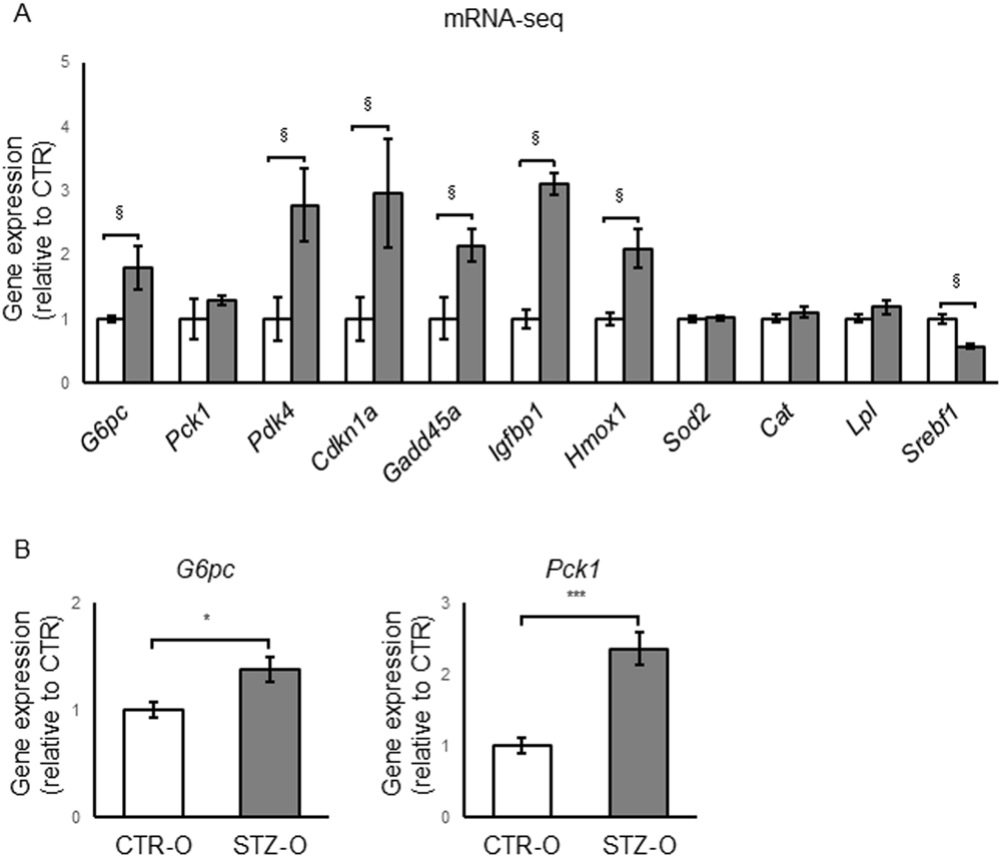
Expression of Foxo1 target genes in the liver of STZ male offspring. Relative expression levels were determined based on mRNA-seq data (n = 3 for CTR and STZ offspring each) (**A**) or by qRT-PCR (n = 8 for CTR and STZ offspring each) (**B**). The average level in CTR offspring was set as 1. Data represent the mean ± SE. ^§^FDR < 0.05, *P < 0.05, ***P < 0.001, N.S. not significant.

Foxo transcription factors are involved in glucose and lipid metabolisms^18^. Both loss- and gain-of-function experiments have established involvement of Foxo1 in hepatic glucose production^19–21^. Based on the results of the KEGG pathway analysis, we speculated that alterations in the Foxo1 pathway may be related to the IGT observed in STZ male offspring. When we investigated the mRNA-seq results in greater detail, several genes known to be upregulated by Foxo1 (*G6pc*, *Pdk4*, *Cdkn1a*, *Gadd45a*, *Igfbp1*, and *Hmox1*)^19,20,22–30^ were upregulated, and a gene known to be downregulated by Foxo1 (*Srebf1*)^31^ was downregulated in STZ male offspring (Fig. 5A). Among the Foxo1 target genes, glucose 6 phosphatase catalytic subunit (*G6pc*) and phosphoenolpyruvate carboxykinase 1 (*Pck1*) are crucial gluconeogenetic enzymes in the liver, which may contribute to the increase in the blood glucose level^21^. Both mRNA-seq and qRT-PCR showed statistically significant upregulation of *G6pc* (Fig. 5A,B). In contrast, while mRNA-seq did not show statistically significant upregulation of *Pck1* in STZ male offspring, higher expression of this gene was clearly detected by qRT-PCR. We also investigated the expression of the Foxo1 target genes in the liver of STZ female offspring and, although several genes were upregulated, *G6pc* and *Pck1* were not (Supplementary Fig. 2).

### Effect of maternal diabetes on Foxo1, Akt, and Erk1/2 phosphorylation

**Figure 6:**
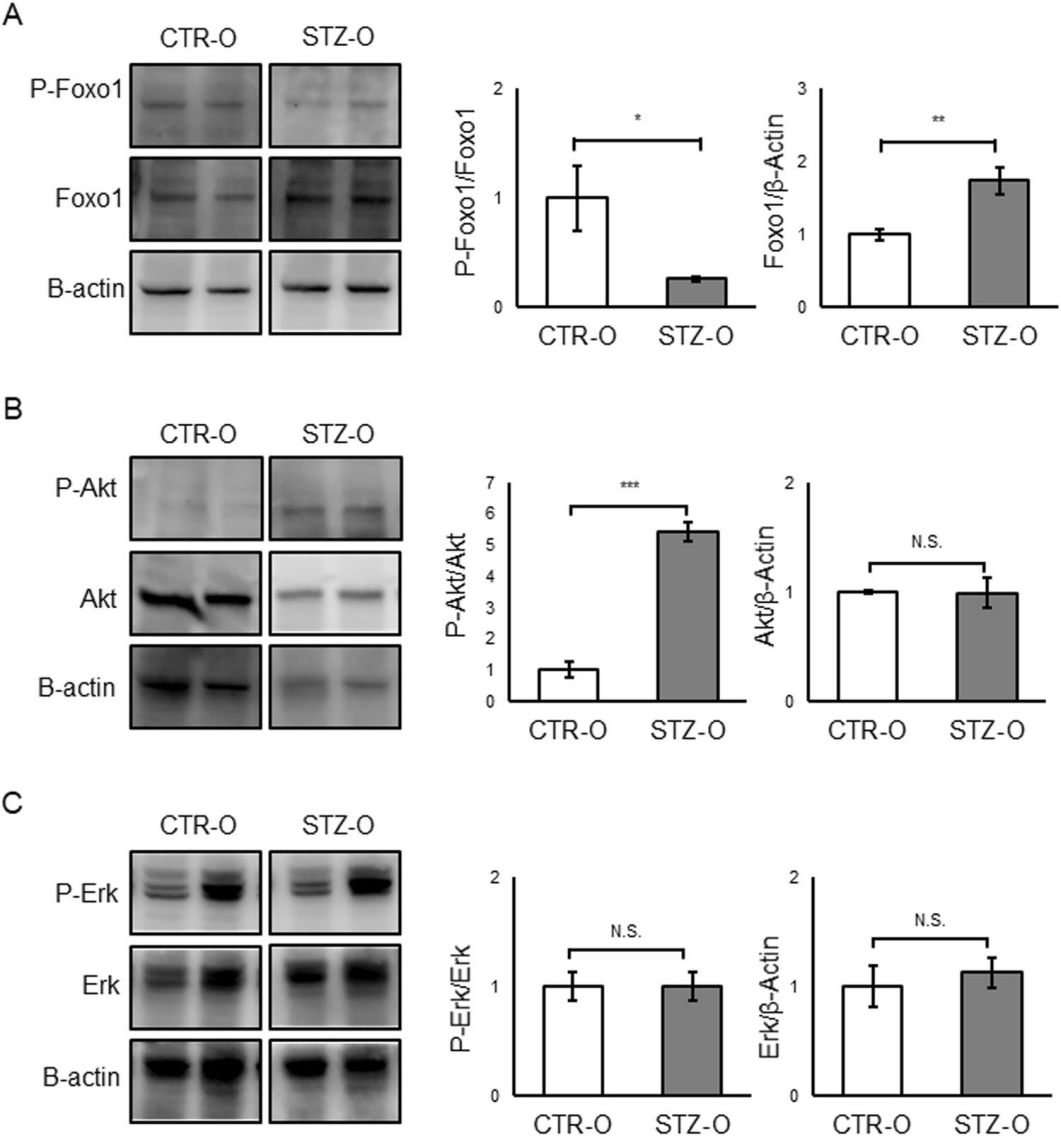
Phosphorylation of Foxo1, Akt, and Erk in the liver of STZ male offspring. Western blotting was performed using antibodies against phospho-Foxo1 and Foxo1 (**A**), phospho-Akt and Akt (**B**), and phospho-Erk and Erk (**C**). An anti-β-actin antibody was used for normalisation. Total protein was obtained from the liver of offspring at 26–27 weeks (n = 4 for each CTR and STZ offspring). The average level in CTR offspring was set as 1. Data represent the mean ± SE. *P < 0.05, **P < 0.01, ***P < 0.001, N.S. not significant. Full-length blots are presented in Supplementary Fig. 4.

Because phosphorylation of Foxo1 at Ser256 is a critical modification that downregulates the activity of this transcription factor^32^, we next investigated the state of this modification in the liver of STZ male offspring at 26–27 weeks. We found that, while the total protein level of Foxo1 was increased in STZ male offspring, the phosphorylation level of this protein was decreased significantly (Fig. 6A). This result suggests enhanced Foxo1 activity that is consistent with the above-described alterations in the Foxo1 target genes. Because Foxo1 phosphorylation is catalyzed by PI3K/protein kinase B (Akt)^33^, we next investigated the phosphorylation state of Akt. Contrary to our expectation, we detected significantly increased phosphorylation of Akt in the liver from STZ male offspring (Fig. 6B). The Ras/Mek/extracellular signal-regulated kinase (Erk) pathway is another important insulin signalling pathway^34^, and Erk signalling is known to be associated with subcellular localisation of Foxo1^35^. We therefore investigated the state of Erk 1/2 phosphorylation at Thr202/Thy204 and found that it was unchanged in the liver of STZ male offspring (Fig. 6C), suggesting that insulin signalling was not activated. In contrast to STZ male offspring, female offspring showed a decreased Foxo1 protein level and an increased phosphorylation level (Supplementary Fig. 3A). The phosphorylation states of Akt and Erk1/2 were not significantly altered in female offspring (Supplementary Fig. 3B,C).

## Discussion

We investigated the effect of poorly controlled maternal diabetes during pregnancy and lactation on the phenotype and pathways related to nutrient metabolism of the exposed offspring. We found that, as reported previously, the offspring have a lower body weight^10,12^ and IGT^9,11,13^, although the phenotypes are not necessarily common to all models, probably because of the species, genotypes, and/or employed experimental conditions. Interestingly, both defects were more pronounced in male offspring. A similar sex-dependent difference in IGT was reported in a previous study^11^, and our results suggest the need for special care for the sex of the offspring exposed to maternal diabetes.

Previous reports have suggested that impaired insulin secretion^9,11^ or insulin resistance^13^ are the cause of IGT in offspring exposed to maternal diabetes. However, our STZ offspring showed no significant change in islet morphology or their fasting insulin level. In addition, the expression of genes related to islet development and functions was not significantly altered in STZ male offspring. Only *Pdx1* showed rather increased expression in female offspring. Several studies have shown that the liver is an organ involved in IGT of animals exposed to s diabetic environment or altered nutrient availability in utero^13,15^. Furthermore, Holmans *et al*. found that STZ offspring have insulin resistance characterised by decreased insulin responsiveness of the liver and peripheral tissues^12^. Considering these points, we focused on the liver of STZ offspring in subsequent molecular analyses.

We identified DEGs by mRNA-seq in the liver and again found a sex difference. In fact, the effect of sex was stronger than that of maternal diabetes. It is possible that the differences arise from the different levels of estrogen in males and females, as this sex hormone is known to regulate glucose and lipid metabolism in multiple organ system, including the liver^36^. We therefore performed all subsequent experiments separately in males and females. Interestingly, some pathways involved in nutrient metabolism, such as the “Foxo signaling pathway” and “PPAR signaling pathway”, were enriched in the DEGs of male offspring, and those such as the “AMPK signaling pathway” and “fatty acid metabolism pathway”, in addition to the “PPAR signaling pathway”, were enriched in the DEGs of female offspring. The altered expression of the multiple Foxo1 target genes, including *G6p*c and *Pck1*, indicated an elevated Foxo1 transactivation activity in the liver of STZ male offspring. Similar upregulation of *G6p*c and *Pck1* was previously reported in a rat intrauterine growth retardation model^16^. Consistent with the findings, the protein level of Foxo1 was increased and its phosphorylation was decreased. Because *G6pc* and *Pck1* are crucial gluconeogenetic enzymes^37–39^, their increased expression may result in increased glucose production, eventually leading to IGT. Consistent with this idea, a previous study has reported increased glucose production in the insulin-resistant liver of STZ offspring^12^.

In contrast, *G6pc* and *Pck1* were not significantly upregulated in the liver of STZ female offspring. Furthermore, the amount and phosphorylation level of Foxo1 suggested a reduction in the Foxo1-transactivating activity. Therefore, we cannot explain the IGT in STZ female offspring by altered Foxo1 activity. While the DEGs of STZ female offspring were enriched for AMPK signalling pathway, which is known to suppress gluconeogenesis through repression of gluconeogenetic enzymes^40^, we did not observe alterations in their expression. Thus, how the IGT occurs in STZ female offspring remains unknown.

Although Akt is the major enzyme responsible for Foxo1 phosphorylation at Ser256^33^, we found that phosphorylation of Akt itself is rather increased in STZ male offspring, suggesting increased Akt activity. This indicates that decreased Foxo1 phosphorylation is primarily independently of Akt phosphorylation status. The increased phosphorylation of Akt and increased protein level of Foxo1 may be the compensative consequences of the decreased phosphorylation of Foxo1^41^. Thus, it is still unclear how maternal diabetes leads to the decreased phosphorylation of Foxo1 in the liver of STZ male offspring. Because additional factors that regulate the localisation and activity of Foxo1 were identified recently^35,42,43^, further studies of all regulatory factors, including the newly identified regulatory factors, are needed to clarify the molecular mechanism underlying the decreased phosphorylation and increased activity of Foxo1.

In summary, our study revealed that poorly controlled maternal diabetes upregulates gluconeogenesis genes through increased expression and reduced phosphorylation of Foxo1 in the liver of STZ male offspring. This may in part contribute to the IGT observed in STZ male offspring. Understanding the precise mechanisms of these events may be important to identify specific risk groups and develop strategies to prevent type 2 diabetes in future generations.

## Methods

### Animals

Seven-week-old female C57BL/6G mice were purchased from Charles River Laboratories. (Yokohama, Japan). All mice were kept under specific pathogen-free conditions, fed with normal chow diet (CA-1; CLEA Japan, Tokyo, Japan), and had free access to water. At 8 weeks, they were divided into two groups. In one group, diabetes was induced by injecting mice with STZ (Sigma, St. Louis, MO, USA) in 0.05 mol/l citrate buffer (pH 4.5) at a dose of 100 mg/kg body weight. Mice with a blood glucose level > 250 mg/dl were considered diabetic. Mice of the other group were injected with citrate buffer, and those with a blood glucose level of < 200 mg/dl were used as CTR mice. Both diabetic and CTR female mice were crossed with healthy JF1/Ms male mice. We obtained 119 and 182 offspring from 16 STZ and 23 CTR mothers, respectively. The STZ and CTR offspring were fed with milk from their own mothers and weaned at 3 weeks. On the day of weaning, we measured the blood glucose levels of the STZ-treated mothers again and used offspring from diabetic mothers only (blood glucose level > 250 mg/dl). After weaning, they were maintained with normal chow diet and free access to water. In total, we used 77 STZ offspring (33 males and 43 females) and 111 CTR offspring (54 males and 57 females) for various experiments. Mouse husbandry and all of the mouse experiments were carried out under the ethical guidelines of Kyushu University. The protocols were approved by the Institutional Animal Care and Use Committee of Kyushu University.

### IPGTT

After fasting for 15 hours, a glucose solution was intraperitoneally injected (1 g/kg body weight). Blood was obtained from the tail vein before (0 minutes) and at 15, 30, 60, and 120 minutes after the injection. Glucose levels were measured *by a ONETOUCH UltraVue* (Johnson & Johnson, Tokyo, Japan).

### Serum insulin levels

Blood was collected from the jugular vein after fasting for 15 hours. The blood samples were centrifuged, and sera were used for insulin measurement using an insulin ELISA kit (Morinaga Institute of Biological Science, Yokohama, Japan).

### Histological analysis of islets

The pancreas was removed, fixed in 10% formaldehyde, and embedded in paraffin. Five micrometre-thick sections were stained with haematoxylin and eosin. Every 20^th^ section (males: 20 sections/pancreas; females: 10 sections/pancreas) was viewed using a bright field illumination microscope (BZ-9000; Keyence, Osaka, Japan), and the area of islets per section was measured. For immunostaining, the samples were incubated with guinea pig anti-insulin antibody (ab7842) (Abcam, Cambridge, UK) followed by goat anti-guinea pig IgG (ab6908) (Abcam, Cambridge, UK). Immunostaining images of 5 islets contained in 5 consecutive cross sections were used for quantitative evaluation. Images were saved as TIFF images and converted to grey scale images using Photoshop software (Adobe Systems, San Jose, CA), and staining intensities were quantitatively analyzed using the ImageJ software.

### RNA extraction

Pancreatic islets were isolated using collagenase as described previously^44^. Total RNA was extracted by using an RNA easy mini kit (*Qiagen, Tokyo, Japan*). Total RNA from liver samples was extracted using ISOGEN (Nippon Gene, Tokyo, Japan).

### qRT-PCR

Total RNA was converted into cDNA using a PrimeScript RT Reagent Kit (Takara, Shiga, Japan). mRNA levels were determined by qRT-PCR using a KAPA SYBR FAST qPCR Kit (Kapa Biosystems Massachusetts, USA). Relative expression levels were calculated by the Δ-ΔCT method and normalised to β-actin mRNA levels. The primer sets were: Pdx1 (forward 5′-GAAATCCACCAAAGCTCACG-3′, reverse 5′-TTCAACATCACTGCCAGCTC-3′), Hmf4α7 (forward 5′-CTCCAGTGGCGAGTCCTTA-3′, reverse 5′-CTCACGCTCCTCCTGAAGAA-3′), Hnf1α (forward 5′-GGCCATGGACACCTATAACG-3′, reverse 5′-GCCGCAGACACTGTGACTAA-3′), Slc2a2 (forward 5′-GACGTCAATGGCACAGACAC-3′, reverse 5′-ATCAAGAGGGCTCCAGTCAA-3′), Ins1 (forward 5′-GGAGCGTGGCTTCTTCTACA-3′, reverse 5′-GTGCAGCACTGATCCACAAT-3′), Ins2 (forward 5′-TTTGTCAAGCAGCACCTTTG-3′, reverse 5′-GGTCTGAAGGTCACCTGCTC-3′), Foxo1 (forward 5′-CCAAGGCCATCGAGAGC-3′, reverse 5′-GATTGAGCATCCACCAAGAACT-3′), Foxo3 (forward 5′-GGGGAACTTCACTGGTGCTA-3′, reverse 5′-TGTCCACTTGCTGAGAGCAG-3′) G6pc (forward 5′-CTGTGCAGCTGAACGTCTGT-3′, reverse 5′- GGAGGCTGGCATTGTAGATG-3′), Pck1 (forward 5′-CCTAGTGCCTGTGGGAAGAC-3′, reverse 5′-CCGTTTTCTGGGTTGATAGC-3′), Pdk4 (forward 5′-TTTCTCGTCTCTACGCCAAG-3′, reverse 5′-GATACACCAGTCATCAGCTTCG-3′), Cdkn1a (5′-TTGTCGCTGTCTTGCACTCT-3′, reverse 5′-AATCTGTCAGGCTGGTCTGC-3′), Igfbp1 (forward 5′-AGATCGCCGACCTCAAGAAAT-3′, reverse 5′-CTCCAGAGACCCAGGGATTTT-3′), Cd36 (forward 5′-CAAGCTATTGCGACATGATT-3′, reverse 5′-CGAACACAGCGTAGATAGAC-3′) and β-actin (forward 5′-CCAACCGTGAAAAGATGACC-3′, reverse 5′-CCATCACAATGCCTGTGGTA-3′).

### Transcriptome analysis

Total RNA of 1 μg was used to construct libraries for mRNA-seq. Libraries were prepared using a TruSeq Stranded mRNA Sample Prep Kit (Illumina, San Diego, CA, USA), in accordance with the manufacturer’s instructions, and sequenced on a HiSeq 2500 to generate 51 nt single-end sequence reads. Illumina adaptor sequences and low-quality bases near the 3′ end (quality score: < 20) were trimmed away by TrimGalore (http://www.bioinformatics.babraham.ac.uk/projects/trim_galore). Read tags were aligned to mouse RefSeq transcripts (mm10) using TopHat2^45^. Reads uniquely mapped to gene exons were normalised by total mapped reads and exon lengths (fragments per kilobase of exon per million mapped sequence reads [FPKM]) using Cuffdiff^46^. The biological replicates showed good reproducibility. (The Pearson correlation coefficient calculated using Log_2_(FPKM + 1) was >0.98 for each replicate group.) When the expression level of a gene was 0 in any sample, this gene was excluded from the analysis. When the average expression levels were under 1 in all groups, such genes were also excluded. Thus, 10703 genes remained for analysis.

### KEGG pathway analysis

David 6.8 (https://david.ncifcrf.gov/) was used for KEGG pathway analysis of DEGs identified in STZ male and female offspring.

### Western blot analysis

Liver samples were immediately frozen in liquid nitrogen and stored at −80 °C until use. The samples were defrosted on ice, homogenised in 1% NP40 lysis buffer containing protease inhibitors (Nacalai Tesque, Kyoto, Japan) and phosphatase inhibitors (Nacalai Tesque), and then centrifuged for 15 minutes at 15,000 × *g*. Protein concentrations were examined using Quick start Bradford Dye Reagent (Bio-Rad, Hercules, CA, USA). Total protein (15 ng) was separated on a 4%–20% Mini-PROTEAN TGX Gel (Bio-Rad, Hercules, CA, USA) and transferred to an Immune-Blot PVDF membrane (Bio-Rad, Hercules, CA, USA). After blocking non-specific binding, the membranes were incubated for 1 hour or overnight with antibodies against pan-Akt (#4691), p44/42 MAPK (#4695), β-actin (#4970), phospho-Akt (Ser473) (#4060), phosphor-P44/42 MAPK (Thr202/Thy204) (#4370), phospho-Foxo1 (Ser256) (#9461) (Cell Signaling Technology, Danvers, MA, USA), or Foxo1 (82358) (Thermo Fisher Scientific, Waltham, MA, USA), followed by Goat anti-rabbit IgG (ab6721) (Abcam, Cambridge, UK) as a secondary antibody. Bands were detected using Chemi-Lumi One Ultra (Nacalai Tesque). Signal strength was measured by ImageJ software (https://imagej.nih.gov/ij/).

### Statistical analysis

Data are presented as the mean and standard error (SE) for variables with a normal distribution and as the median and interquartile range for variables with a non-normal distribution. Differences between the two groups were analysed using the Student’s t-test or chi-squared test. Multiple comparisons among groups were performed by one-way analysis of variance with Fisher’s probable least squares difference test for post-hoc analysis. P-values of <0.05 were considered as significant.

## Supplementary information

Supplementary information

Supplementary data

## Data Availability

The raw fastq files generated in this study have been deposited in the NCBI Sequence Read Archives under accession number PRJNA524434.
