# Supplementary material for: Poorly controlled diabetes during pregnancy and lactation activates the Foxo1 pathway and causes glucose intolerance in adult offspring
Source: Sci Rep. 2019 Jul 15;9:10181. doi: 10.1038/s41598-019-46638-2 (PMC6629688; doi:10.1038/s41598-019-46638-2)
Supplement: Supplementary file 1 — Supplementary information [file 41598_2019_46638_MOESM1_ESM.pdf]

Poorly controlled diabetes during pregnancy and lactation activates the Foxo1 pathway and causes glucose intolerance in adult offspring

Yukihiro Inoguchi[1,2,*], Kenji Ichiyanagi[1,3], Hiroaki Ohishi[1], Yasutaka Maeda[4], Noriyuki Sonoda[2], Yoshihiro Ogawa[2,5,6,], Toyoshi Inoguchi[7] and Hiroyuki Sasaki[1,*]

[1]Division of Epigenomics and Development, Department of Molecular and Structural Biology Medical Institute of Bioregulation, Kyushu University

[2]Department of Medicine and Bioregulatory Science, Graduate School of Medical Sciences, Kyushu University

[3]Laboratory of Genome and Epigenome Dynamics, Department of Animal Sciences, Graduate School of Bioagricultural Sciences, Nagoya University

[4]Clinical Research Center for Diabetes, Clinic Masae Minami

[5]Department of Molecular and Cellular Metabolism, Graduate School of Medical and Dental Sciences, Tokyo Medical and Dental University

[6]AMED-CREST, Japan Agency for Medical Research and Development, Tokyo, Japan

[7]FUKUOKA Health Promotion Support Center

*Correspondence and requests for materials should be addressed to Y.I. (e-mail: ykinoguchi@gmail.com) or H.S. (e-mail: hsasaki@bioreg.kyushu-u.ac.jp)

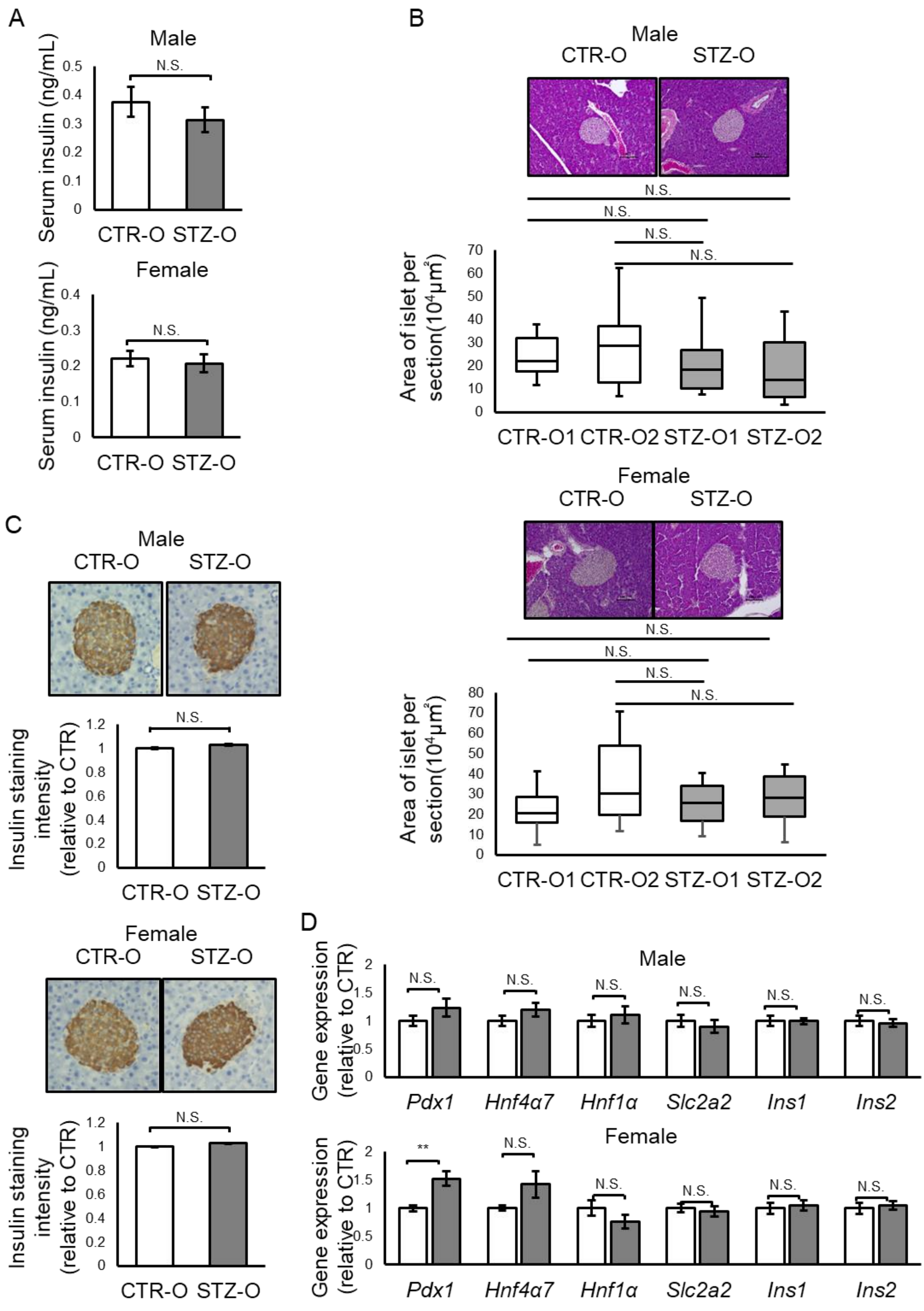

Supplementary Fig. 1. Serum insulin level, islet morphology, and expression of insulin-

related genes. (A) Fasting serum insulin level. The levels were measured after fasting for 15 hours in CTR and STZ offspring of each sex at 25 weeks after birth (n=8 each). (B) Morphology of pancreatic islets. Tissues were obtained from CTR and STZ offspring of each sex at 26–27 weeks (n=2 each). Haematoxylin and eosin staining of representative sections is shown at the top. Box plots show the distribution of the area of islets per section with the median and interquartile ranges. Data were obtained from 20 and 10 sections/pancreas in male and female offspring, respectively, as males have a larger pancreas. (C) Representative immunostaining images of the same pancreatic specimens (n=2 each) with anti-insulin antibody is shown at the bottom. Graphs show the relative insulin staining intensity. Data were obtained from 5 sections/pancreas in both male and female offspring. N.S. not significant. (D) Expression levels of insulin-related genes in islets determined by qRT-PCR. Islets were isolated at 18 weeks (CTR male offspring, n=7; CTR female offspring, n=8; STZ male offspring, n=4; STZ female offspring, n=6). The expression level of β-*actin* was used for normalization. The average level in CTR offspring was set as 1. Data represent the mean ± SE. **P<0.01, N.S. not significant.

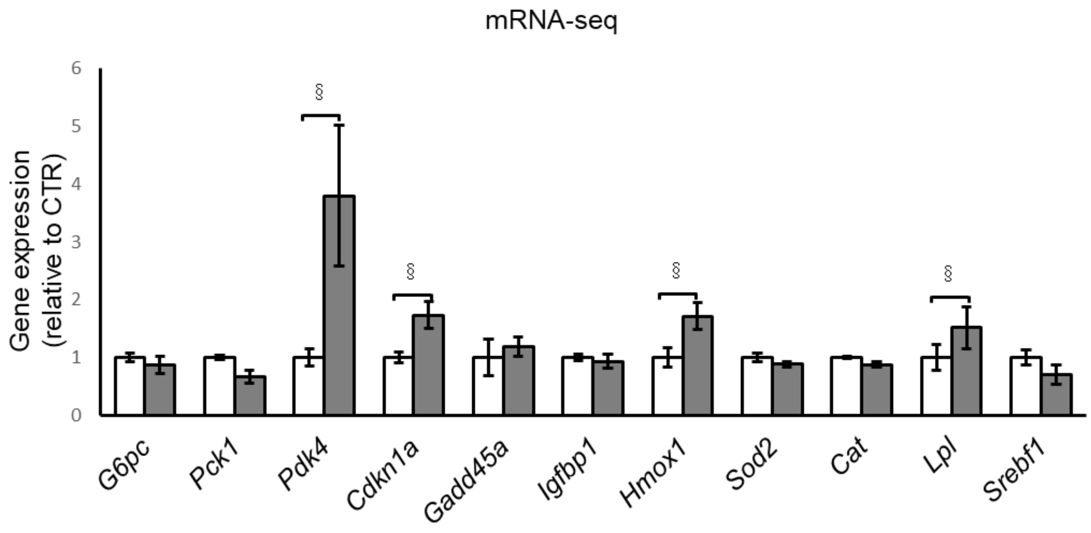

Supplementary Fig. 2. Expression of Foxo1 target genes in the liver of STZ female offspring**.** Relative expression levels were determined using mRNA-seq data (n=3 for CTR and STZ offspring each). The average level in CTR offspring was set as 1. Data represent the mean ± SE. [§]FDR<0.05.

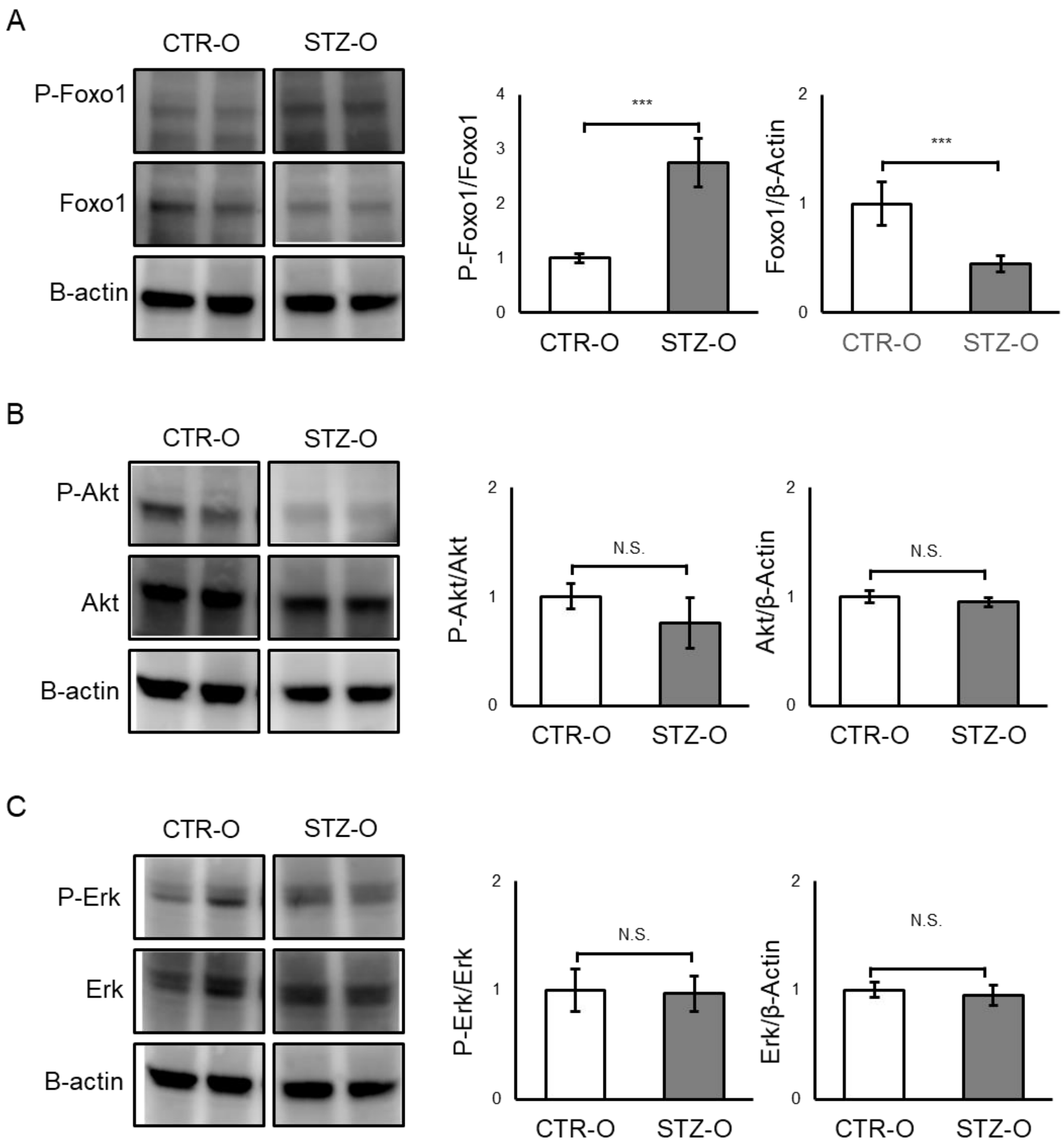

Supplementary Fig. 3. Phosphorylation of Foxa1, Akt, and Erk in the liver of STZ female offspring. Western blotting was performed using antibodies against phospho-Foxo1 and Foxo1 (A), phospho-Akt and Akt (B), and phospho-Erk and Erk (C) (n=4 for CTR and STZ offspring each). Details are described in the **Figure 6** legend. Full-length blots are presented in **Supplementary Fig. 5.**

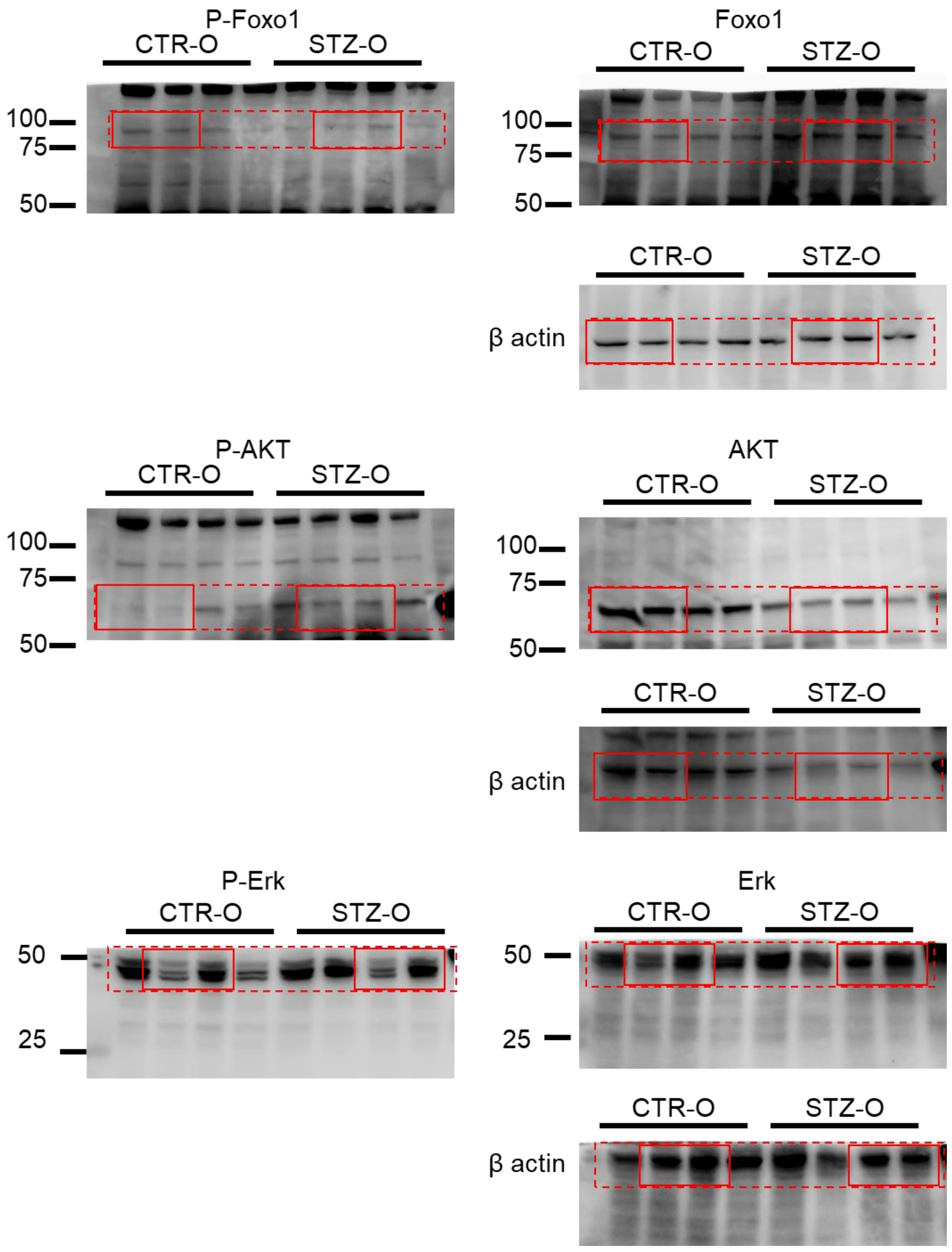

Supplementary Fig. 4. Full-length blots for **Figure 6**. The cropped areas are boxed by red lines.

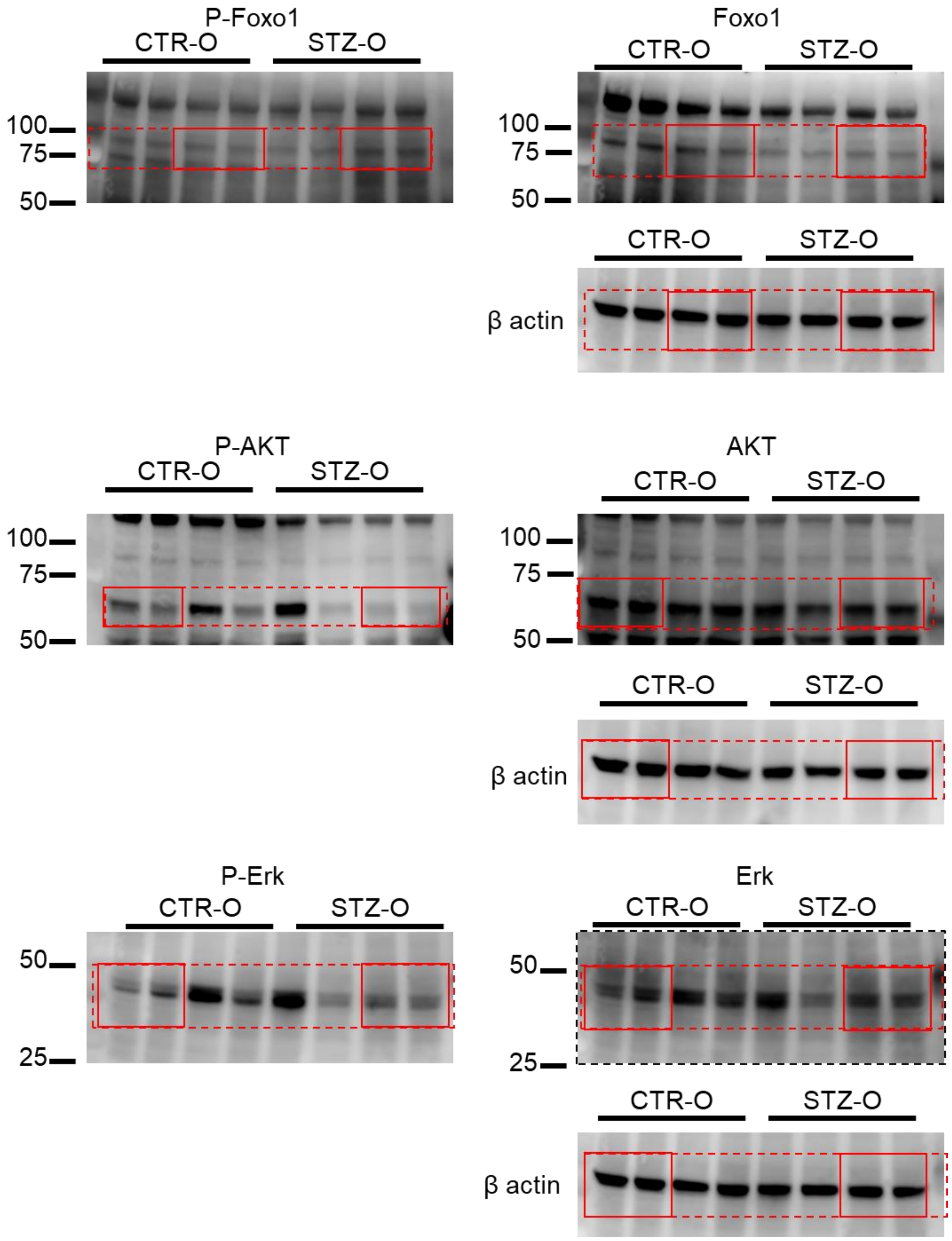

Supplementary Fig. 5. Full-length blots for **Supplementary Fig. 3**. The cropped areas are boxed by red lines.
